# Predictive value of neutrophil-lymphocyte ratio for all-cause mortality in patients with chronic obstructive pulmonary disease: a systematic review and meta-analysis

**DOI:** 10.1186/s12890-025-03677-y

**Published:** 2025-04-29

**Authors:** Li Fang, Jianzhi Zhu, Dandan Fu

**Affiliations:** The Second Affiliated Hospital of ZunYi Medical University, Guizhou, China

**Keywords:** Chronic obstructive pulmonary disease, Neutrophil-to-lymphocyte, Ratio

## Abstract

**Background:**

Chronic obstructive pulmonary disease (COPD) involves inflammation as a key factor influencing its pathology and progression. This meta-analysis sought to assess the prognostic importance of the neutrophil-to-lymphocyte ratio (NLR) in individuals diagnosed with COPD.

**Methods:**

Comprehensive searches were carried out in PubMed, Embase, Web of Science, and the Cochrane Library up to March 2025. All-cause mortality-related data were collected and analyzed. Outcomes were evaluated using odds ratios (ORs) and 95% confidence intervals (CIs).

**Results:**

Following a thorough review of the literature and a rigorous screening process, a total of 24 studies including 18,597 patients were selected for this meta-analysis. The cut-off range of NLR in all included literatures was 1.3 to 16.83. Analysis of categorical variables showed that COPD patients with elevated NLR levels faced a significantly higher all-cause mortality risk compared to those with lower NLR levels (OR: 1.03, 95% CI: 1.01–1.06, *P* = 0.009, *I²* = 89%). For continuous variables, deceased COPD patients exhibited significantly elevated NLR levels compared to survivors (SMD: 1.23, 95% CI: 0.90–1.57, *P* < 0.00001, *I²* = 97%). The subgroup analysis highlighted study design and the timing of NLR measurement as potential contributors to heterogeneity. Subgroup analysis showed that NLR had a better predictive value for disease in AECOPD subgroups.

**Conclusion:**

This meta-analysis demonstrates a correlation between increased NLR levels and heightened all-cause mortality risk in COPD patients. Nevertheless, given the inherent limitations of this study, additional multi-center, prospective clinical trials are essential to confirm these findings.

**Supplementary Information:**

The online version contains supplementary material available at 10.1186/s12890-025-03677-y.

## Introduction

Chronic obstructive pulmonary disease (COPD) is a diverse lung condition characterized by persistent and often worsening airflow obstruction. This disorder results from structural abnormalities in the airways, such as bronchitis or bronchiolitis, and/or alveolar damage observed in emphysema. These alterations lead to ongoing respiratory issues, including breathlessness, chronic coughing, and sputum production (GOLD 2023) [1]. The World Health Organization predicts that by 2030, COPD will cause over 3 million deaths annually, positioning it as the third leading cause of mortality. Increased hospital readmissions and related complications significantly diminish patients’ quality of life and heighten their mortality risk [2–4]. The worldwide prevalence of COPD is projected to rise to 600 million cases by 2050, marking a 23% surge compared to 2020. Moreover, COPD imposes a heavy economic strain on society. Within the European Union, respiratory diseases account for 6% of total healthcare expenditures, with COPD comprising 56% (€38.6 billion) of the costs related to respiratory conditions [5]. For clinical management, inhaled bronchodilators and glucocorticoids remain the cornerstone treatments [6]. To enhance COPD management and alleviate the associated financial burden, identifying reliable biomarkers for early and precise evaluation of short-term and long-term prognoses, along with readmission risks, is critically important.

Airway inflammation represents a crucial pathological characteristic of COPD, intimately tied to its development and progression. Monitoring inflammation in COPD patients involves key biomarkers such as neutrophils, lymphocytes, eosinophils, and C-reactive protein (CRP) [7–12]. Neutrophils significantly contribute to the initial inflammatory response and act as central mediators in COPD, being linked to reduced lung function, impaired gas exchange, and alveolar destruction [13, 14]. In COPD, the imbalance between proinflammatory responses mediated by Th1 and anti-inflammatory responses mediated by Th2 plays a crucial role in maintaining inflammation and advancing disease progression. Patients with COPD show elevated levels and heightened functional activity of CD8 + T cells. These cells primarily infiltrate the lung parenchyma and airways, promoting emphysema by releasing cytotoxic molecules such as perforin and granzyme B, leading to the lysis and apoptosis of structural lung cells [15–17]. Synthesized in the liver, CRP is an acute-phase inflammatory protein strongly associated with late-stage mortality in COPD patients, as evidenced by studies [18, 19]. Additionally, recent findings highlight that the CRP-to-albumin ratio (CAR) correlates with mortality risk, with elevated CAR levels predicting a higher likelihood of 5-year mortality [20]. Terminal granulocytes like eosinophils serve as both immune effector and inflammatory cells. Their levels are commonly utilized as inflammation markers for prognosis and treatment assessment in COPD management [21]. Furthermore, studies suggest that eosinophil counts can predict mortality in patients with acute exacerbations of AECOPD [22, 23]. COPD is characterized by an inflammatory response dominated by neutrophils and lymphocytes, releasing inflammatory mediators and interacting with structural cells of the airways and lung parenchyma [24, 25].

To further investigate the significance of neutrophil-to-lymphocyte ratio (NLR) in predicting all-cause mortality in individuals with COPD, we collected the latest and most comprehensive data for an in-depth analysis. This study seeks to offer theoretical foundations for designing a clinical risk prediction model for COPD, facilitating the accurate identification of high-risk patients and promoting timely, evidence-informed therapeutic interventions.

## Materials and methods

### Data sources and searches

This study was conducted in adherence to the PRISMA2020 guidelines as outlined in the statement [26] and its protocol was recorded in the International Prospective Systematic Evaluation Registry (PROSPERO: CRD42024590075). A comprehensive search was performed across several databases, including PubMed, Embase, Web of Science, and the Cochrane Library, with data retrieved up to March 2025. The search incorporated MeSH terms such as ‘Neutrophil’, ‘Leukocytes, Polymorphonuclear’, ‘Chronic Obstructive Pulmonary Diseases’, ‘COPD’, ‘Decline, Mortality’, and ‘Mortality, Excess’, with an expanded scope where applicable. Details of the search strategies can be found in Supplementary File 1.

### Outcomes

In this meta-analysis, all-cause mortality served as the outcome measure, defined as death from any cause occurring throughout the follow-up period.

### Identification criteria

Studies meeting the following criteria were included: (i) individuals diagnosed with chronic obstructive pulmonary disease (COPD); (ii) research reporting inflammation markers, such as neutrophil-to-lymphocyte ratio (NLR); (iii) studies presenting results from multivariate analyses, including odds ratios (OR) or standardized mean differences (SMD) with corresponding 95% confidence intervals (CI). Excluded were: (i) abstracts from conferences, letters to editors, case reports, and studies involving animals; (ii) research articles without accessible full texts; and (iii) publications in languages other than English. Two reviewers independently screened studies according to these criteria, resolving any disagreements by reaching a consensus.

### Data extraction and quality assessment

Information such as the year of publication, the country of the first author, study design, sample size with sex distribution, age, timing of sample collection, follow-up period, inflammatory markers (e.g., NLR), and outcomes were obtained from the selected studies. The Newcastle-Ottawa Scale (NOS) was used to evaluate the quality of these studies, with a maximum score of 9 and scores of 6 or above indicating high quality [27]. Furthermore, the processes of data extraction and quality assessment were independently carried out by two reviewers.

### Statistics analysis

The odds ratio (OR) for continuous variables and the standardized mean difference (SMD) for categorical variables, together with their corresponding 95% confidence intervals (CIs), were computed to evaluate the all-cause mortality ratio among COPD patients. To assess heterogeneity, Cochran’s Q test and the Higgins I² statistic were utilized [28]. Significant heterogeneity was indicated when *I*^*2*^ > 50% or *P* < 0.1. All data analyses were performed using the random effects model. Subgroup and sensitivity analyses were carried out to ensure the reliability of the findings related to overall survival (OS) and progression-free survival (PFS). Funnel plots, along with Egger’s and Begg’s tests, were used to examine publication bias. Statistical significance was defined as *P* < 0.05. The statistical analyses were performed using Review Manager 5.4 and STATA version 15.0 software.

## Results

### Study screening procedure

**Fig. 1:**
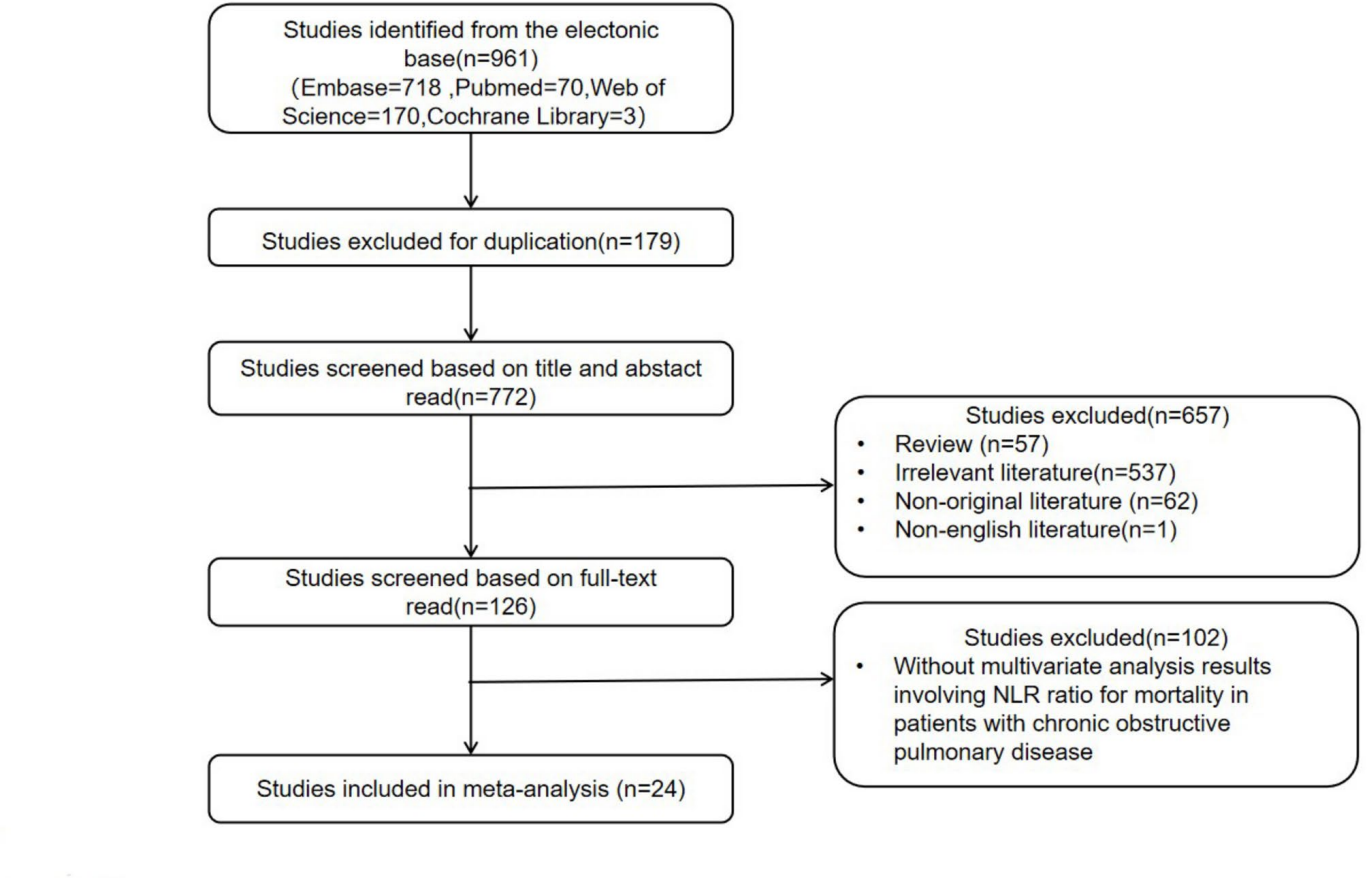
Flow chart of literature screening

From electronic databases, a total of 961 studies were identified: 718 from Embase, 70 from PubMed, 170 from Web of Science, and 3 from the Cochrane Library. After removing 179 duplicates, 772 studies underwent title and abstract screening. During this process, 57 review articles, 537 studies deemed irrelevant, 62 non-original studies, and 1 non-English study were excluded. Following this, full-text analysis was conducted for 126 studies, which resulted in the exclusion of 102 studies that did not report multivariate analysis findings on the association between the NLR ratio and all-cause mortality in chronic obstructive pulmonary disease patients. Consequently, 24 studies were included in the meta-analysis (Fig. 1).

### Features of included studies

The selected studies, published from 1999 to 2025, encompassed a total of 18,597 participants. Among these, ten studies were carried out in China, two in Turkey, and another three in Egypt. The remaining studies were conducted in various countries, including Süreyyapaşa (Iran), Japan, Greece, Colombia, the United States, India, and Australia. The follow-up periods ranged between 1 day and 2 years. The cut-off range of NLR in all included literatures was 1.3 to 16.83. Table 1 provides detailed information regarding the studies included in the analysis [24–25, 29–50]. Each study was evaluated using the Newcastle-Ottawa Scale, with bias risk scores between 7 and 9, signifying a low risk of bias, as shown in Table 1.

**Table 1:** Included studies

Study	Year	Country	Study design	Population	Patients (*n*)	Gender		Follow -up	NLR threshold
						Male	Female		
Aksoy et al. [29]	2018	Süreyyapaşa	Retro	AECOPD	2727	1867	860	28d	15
Alkhayat et al. [30]	2021	Egypt	pro	COPD	38	38	0	2Y	1.3
Ardestani et al. [31]	2021	Iran	Retro	AECOPD	829	555	274	NA	6.9
Duman et al. [32]	2015	Japan	Retro	COPD	1704	146	1558	6 M	7
Dwedar et al. [24]	2018	Egypt	pro	AECOPD	50	38	12	NA	6.24
Feng et al. [33]>	2023	China	Retro	AECOPD	503	489	14	90d	14.17
Galani et al. [34]	2021	Greece	Retro	AECOPD	127	86	41	28d	NA
Gayaf et al. [35]	2021	Turkey	pro	COPD	141	115	26	90d	NA
Gómez-Rosero et al. [36]	2021	Colombia	Retro	COPD	619	319	300	NA	5
Hu et al. [37]	2023	US	Retro	COPD	1715	966	749	NA	8.13
Karkra et al. [38]	2023	India	Retro	AECOPD	500	436	64	NA	14.83
Kumar et al. [39]	2017	Australia	Retro	AECOPD	181	88	93	90d	NA
Liu et al. [25]	2019	China	Retro	AECOPD	622	305	317	3 M	4.19
Luo et al. [40]	2021	China	Retro	AECOPD	533	355	178	28d	6.74
Peng et al. [41]	2022	China	Retro	AECOPD	494	248	246	30d	NA
Shao et al. [42]	2023	China	Retro	AECOPD	4235	3137	1098	1y	4.43
Sunnetcioglu, et al. [43]	2022	Turkey	Retro	COPD	134	83	51	30d	NA
Xiong et al. [44]	2017	China	Retro	COPD	368	116	252	24 M	3.3
Yao et al. [45]	2017	China	Retro	AECOPD	303	200	103	15d	6.24
Yao et al. [46]	2021	China	Retro	AECOPD	146	109	37	28d	16.83
Yu et al. [47]	2020	China	Retro	AECOPD	695	576	119	NA	NA
Fayiad et al. [48]	2024	Egypt	Retro	COPD	80	79	1	NA	NA
Liao et al. [49]	2024	China	Retro	AECOPD	619	516	103	NA	NA
Liu et al. [50]	2025	US	Retro	COPD	1234	677	577	24 h	NA

### NLR for predicting mortality

**Fig. 2:**
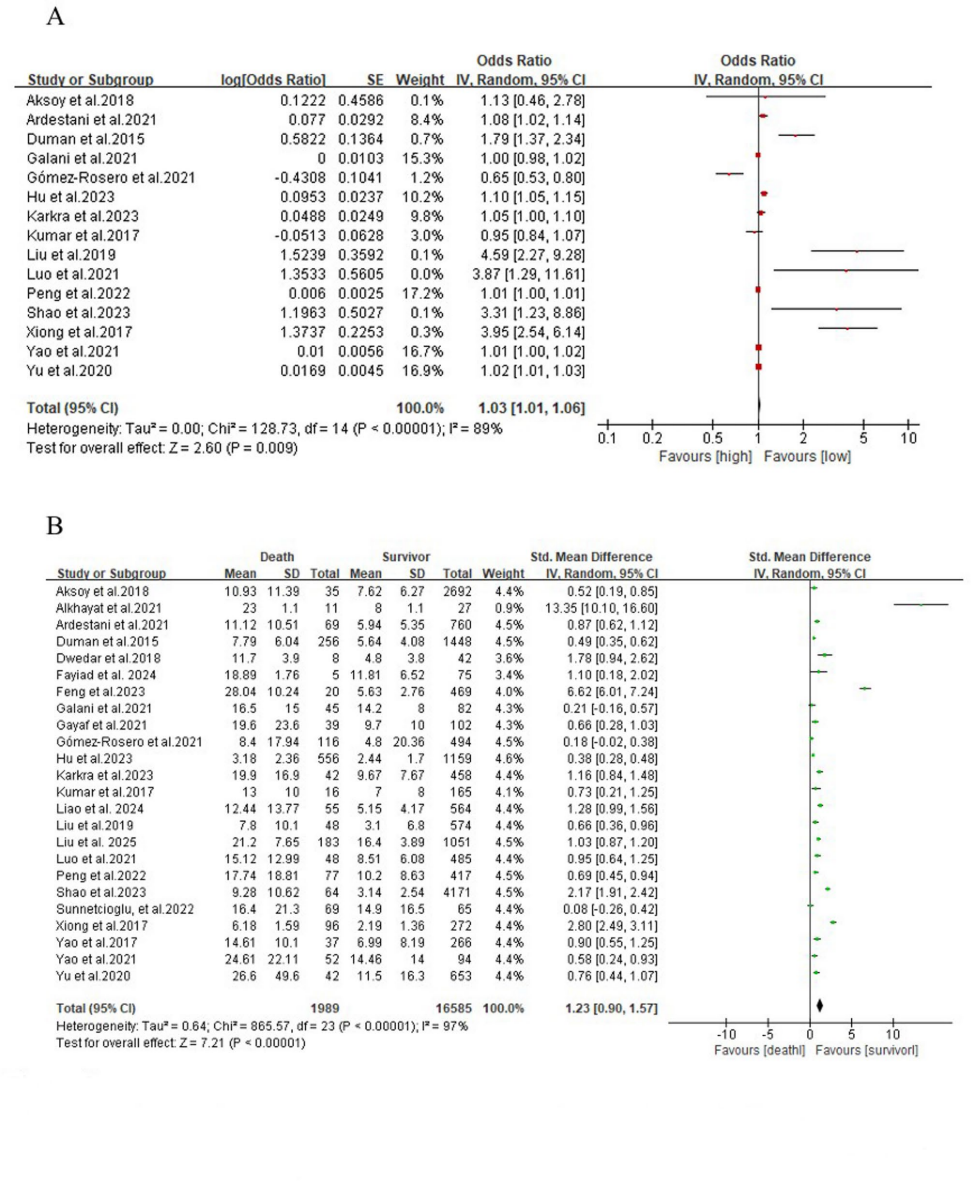
A forest plot predicted mortality in COPD patients, demonstrating the correlation between the NLR and the Odds Ratio (**A**), alongside mortality outcomes for both deceased and surviving patients (**B**)

A total of 15 studies were analyzed for categorical variables. The results of the meta-analysis revealed that COPD patients exhibiting a high NLR faced a notably increased all-cause mortality risk compared to those with a low NLR (OR: 1.03, 95% CI: 1.01–1.06, *P* = 0.009, *I²* = 89%) (Fig. 2A). Specifically, the all-cause mortality risk for COPD patients with a high NLR was observed to be 1.03 times greater than for patients with a low NLR.

For continuous variables, a total of 24 studies were analyzed in the meta-analysis. The findings demonstrated that the NLR levels were markedly elevated in COPD patients who succumbed compared to those who survived (SMD: 1.23, 95% CI: 0.90–1.57, *P* < 0.00001, *I²* = 97%) (Fig. 2B).

### Sensitivity analysis

**Fig. 3:**
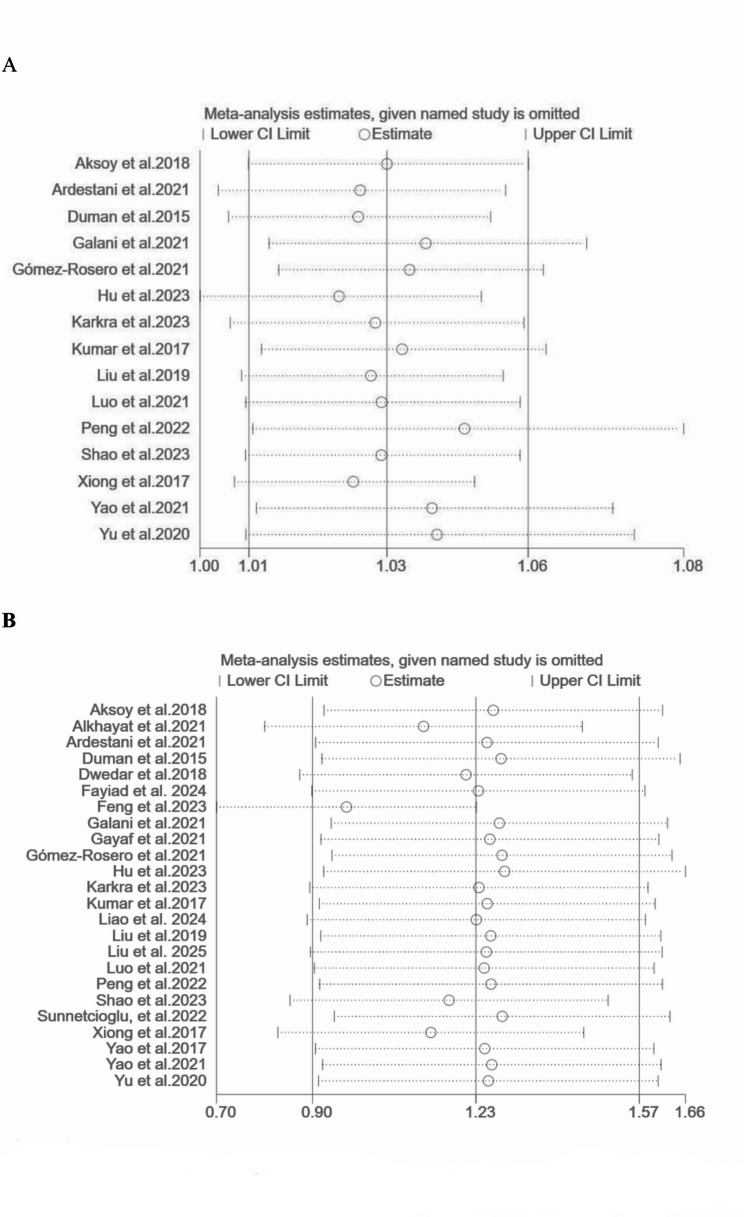
A sensitivity analysis was conducted for categorical mortality variables (**A**) and continuous mortality variables (**B**)

In this research, sensitivity analyses were performed to evaluate all-cause mortality outcomes for both continuous and categorical variables, aiming to examine the impact of individual studies on the overall findings. Regarding the categorical mortality variable, the exclusion of data from Hu 2023^37^ resulted in a shift in the outcomes from significant to nonsignificant (Fig. 3A), highlighting potential instability. Conversely, for continuous variables, the exclusion of any single study did not affect the statistical significance of the overall results, demonstrating the robustness of the indicator (Fig. 3B).

### Subgroup analysis

A subgroup analysis was performed considering factors such as study design, follow-up duration, geographic region, disease stage, sample size, age, and NLR. No significant association between NLR and mortality was detected in subgroups with located in the Africa, Oceania, European and Oceania, COPD, sample size, or including individuals younger than 70 years. Conversely, significant associations between NLR and all-cause mortality were identified in the other subgroups. Regarding the subgroup analysis of continuous variables, NLR values exhibit a significant relationship with COPD all-cause mortality in the all subgroups. Additional details on subgroup analyses can be found in Table 2.

**Table 2:** Subgroup analysis of mortality in COPD

Subgroup	Mortality(Classified variable)				Mortality(Continuity variable)			
	Study	OR [95%CI]	*P* value	I^2^	Study	OR [95%CI]	*P* value	I^2^
***Total***	15	1.03 [1.01, 1.06]	0.009	89%	24	1.23[0.90–1.57]	<0.00001	97%
***Follow-up***								
≥ 90 days	6	2.14[1.20, 3.83]	<0.00001	93%	8	2.70[1.61,3.8]	<0.00001	99%
< 90 days	3	1.01[0.99,1.02]	0.05	68%	9	0.66[0.42,0.89]	<0.00001	81%
***Region***								
Asia	10	1.04 [1.01,1.06]	0.005	91%	15	1.35 [0.86,1.85]	<0.00001	98%
Europe and Americas	3	1.00 [0.86, 1.16]	1	94%	4	0.46 [0.07, 0.86]	0.02	95%
Africa	1	1.13 [0.46, 2.78]	0.79	/	4	3.14 [1.13, 5.16]	0.002	95%
Oceania	1	0.95 [0.84, 1.07]	0.41	/	1	0.21 [-0.16, 0.57]	0.26	/
Population								
COPD	4	1.44[0.89,2.31]	0.14	96%	9	1.06[0.58,1.54]	<0.00001	98%
AECOPD	11	1.02[1.00,1.03]	0.04	77%	15	1.30[0.81,1.78]	<0.00001	97%
Sample size								
≥ 500	9	1.11[1.00, 1.23]	0.05	90%	13	1.26[0.84,1.69]	<0.00001	98%
<500	6	1.01[0.99,1.04]	0.27	88%	11	1.28[0.65,1.91]	<0.00001	96%
Mean age								
≥ 70y	13	1.03[1.00, 1.06]	0.02	91%	17	1.18[0.78,1.58]	<0.00001	98%
<70y	2	1.03[0.96,1.11]	0.38	84%	6	1.47[0.59,2.35]	0.0002	93%
NLR cut-off								
≥ 7	5	1.09[1.01,1.17]	0.03	87%	/	/	/	/
<7	5	1.91[1.,10, 3.34]	0.02	95%	/	/	/	/

OR, odds ratio; CI, confidence interval

### Publication bias

**Fig. 4:**
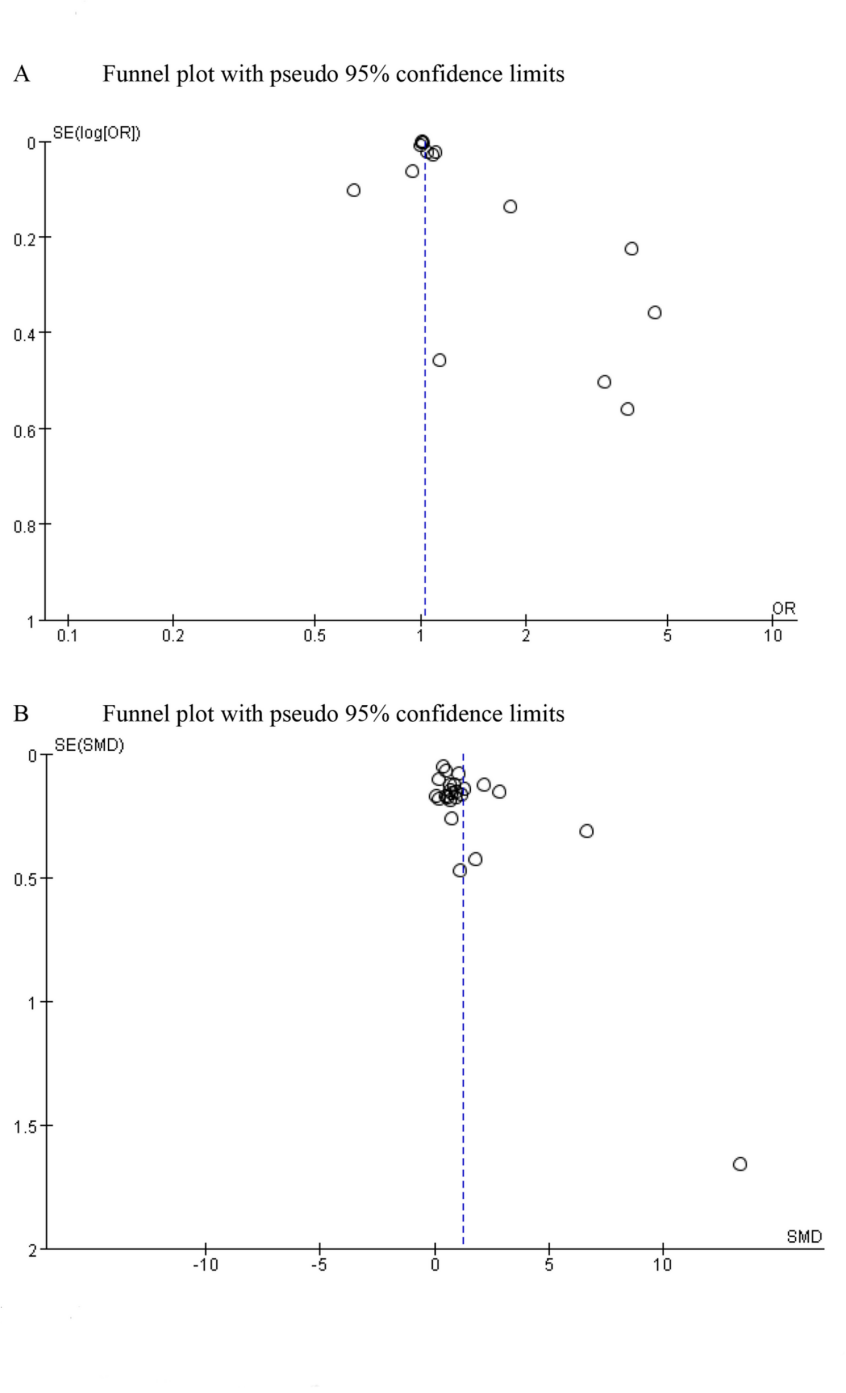
The funnel plot was used to assess publication bias for categorical mortality variables (**A**) and continuous mortality variables (**B**)

Publication bias was evaluated for both categorical and continuous mortality variables. The results from the funnel plot suggested symmetry in the categorical mortality variables, and Egger’s test did not provide evidence of publication bias (*P* = 0.027) (Fig. 4A). Likewise, the funnel plot for continuous mortality variables showed symmetry, with Egger’s test also indicating no publication bias (*P* = 0.015) (Fig. 4B).

## Discussion

In AECOPD patients, common biomarkers used to assess the inflammation severity and predict prognosis include CRP, interleukin-6, and calcitonin. However, these markers can be influenced by multiple factors such as comorbidities like cancer and heart failure, as well as age, and are often expensive. The NLR, which measures the ratio of neutrophils to lymphocytes, is a simple, rapid, and cost-effective test that can be easily performed with routine bloodwork. Research has shown that NLR is a more reliable predictor of all-cause mortality risk in AECOPD patients compared to CRP [7, 29, 30, 44]. Moreover, higher NLR values have been associated with greater 30-day and 90-day mortality rates, indicating a direct correlation between elevated NLR and increased mortality [35, 37].

In our meta-analysis, which encompassed 18,597 patients, we assessed the prognostic significance of the NLR ratio for all-cause mortality in individuals with COPD. The findings demonstrated that COPD patients exhibiting a high NLR were at a considerably greater risk of all-cause mortality compared to those with a low NLR. To investigate the sources of heterogeneity, we conducted a sensitivity analysis. Removing any study from the continuous variable analysis did not alter the overall statistical significance, suggesting the stability of the indicators. Further research involving categorical variables is necessary for clearer understanding. No publication bias was observed in either article type, reinforcing the credibility of our results. In addition, a subgroup analysis was performed to explore heterogeneity further. The analysis revealed that the predictive power of NLR differed across various subgroups. It was particularly relevant to continuous variables and to patients with a follow-up duration of ≥ 90 days, those from Asia, those with a sample size ≥ 500, and those with an average age of ≥ 70 years in the case of categorical variables. Statistics indicate that approximately 384 million people worldwide are affected by COPD, with about 100 million cases in China [51], highlighting the high prevalence of COPD in Asia, consistent with our results. The subgroup analysis also suggested that sample size, disease stage and age might influence the NLR’s predictive value. However, due to the limited number of studies, no significant differences were found. Additional studies are needed to validate whether these factors influence the predictive value of NLR.

In patients with COPD, neutrophils accumulate on the airway endothelial cells and migrate toward the respiratory tract, influenced by chemokines like interleukin-8 and leukotriene-B4. This migration triggers an increase in neutrophil count, which leads to the release of reactive oxygen species and proteolytic enzymes. These factors contribute to the collapse of alveoli and the development of emphysema. At the same time, lung tissue in individuals with COPD experiences adaptive immune responses, driven by triggers such as tobacco, bacteria, viruses, and byproducts of extracellular matrix breakdown. A significant rise in the number of CD8 + T lymphocytes, key immune cells, further exacerbates airflow limitation and emphysema. The activation of CD8 + T cells results in the release of perforin and granulysin, leading to the apoptosis of structural cells. The neutrophil-to-lymphocyte ratio (NLR) has been identified as a prognostic marker for a range of cancers, including pancreatic, esophageal, melanoma, colorectal, diffuse large B-cell lymphoma, and non-small cell lung cancer. Elevated NLR also serves as an indicator of poor prognosis in chronic conditions such as chronic kidney disease, coronary artery disease, appendicitis, systemic lupus erythematosus, and cystic fibrosis [25, 40]. This meta-analysis incorporates data from 24 studies, reinforcing the significant predictive value of NLR for COPD patient prognosis, aligning with prior research outcomes.

Our meta-analysis does have some limitations. To begin with, the majority of the eligible studies were conducted in Asia, which may limit the ability to generalize our findings to other regions, such as Europe, Africa, and the Americas. Therefore, further validation of the NLR’s role in predicting mortality risk in COPD patients from non-Asian populations is needed. Another limitation is that several studies included in the analysis were retrospective, which could introduce confounding factors that might affect the reliability of the results. Moreover, the differences in NLR cut-off values across studies (ranging from 1.3 to 16.83) create additional limitations. To improve the reliability and comparability of future research, it is essential to establish standardized NLR cut-off values. Furthermore, this study focused solely on assessing the predictive value of the NLR for all-cause mortality in COPD patients. Future research directions should prioritize investigating respiratory-specific mortality, AECOPD and pneumonia-related risks in this population. Despite these limitations, our meta-analysis, which involves the largest sample size to date compared with previous studies with sample sizes 716–9706 patients [52–55], indicates that NLR is linked to mortality in COPD patients. Subgroup analyses reinforce the finding that NLR has stronger predictive value in continuous variable literature and in populations with follow-up durations of 90 days or more, from Asia, with sample sizes greater than 500, and average ages of 70 years or older in categorical variables. Our results are also consistent with most of the previous studies, offering updated theoretical support and evidence for developing risk models to predict all-cause mortality in COPD patients.

## Conclusion

NLR is considered a crucial biomarker for assessing the risk of all-cause mortality in COPD patients, as an elevated NLR is strongly associated with a higher risk of death. According to subgroup analyses, variables such as geographic location and age could impact the predictive significance of NLR. The study’s limitations, such as its retrospective nature, disease stage and potential selection bias from regional factors, and possible heterogeneity, have been noted. To improve the findings, extensive, multicenter, high-quality clinical trials with long-term follow-up are required.

## Electronic supplementary material

Below is the link to the electronic supplementary material.

Supplementary Material 1

Supplementary Material 2

## Data Availability

The data to support the findings of this study are included within the article.
