# Supplementary material for: Predictive value of neutrophil-lymphocyte ratio for all-cause mortality in patients with chronic obstructive pulmonary disease: a systematic review and meta-analysis
Source: BMC Pulm Med. 2025 Apr 29;25:206. doi: 10.1186/s12890-025-03677-y (PMC12039089; doi:10.1186/s12890-025-03677-y)
Supplement: Supplementary file 2 — Supplementary Material 2 [file 12890_2025_3677_MOESM2_ESM.pdf]

## Supplementary File 1.

Pubmed:70

| | | | | | |
|---|---|---|---|---|---|
| #14 | | | Search: **(((((((((((((((((((((((((((((((((((((((((((Mortalities[Title/Abstract]) OR (Mortality Rate[Title/Abstract])) OR (Mortality Rates[Title/Abstract])) OR (Rate, Mortality[Title/Abstract])) OR (Death Rate[Title/Abstract])) OR (Death Rates[Title/Abstract])) OR (Rate, Death[Title/Abstract])) OR (Case Fatality Rate[Title/Abstract])) OR (Case Fatality Rates[Title/Abstract])) OR (Rate, Case Fatality[Title/Abstract])) OR (Rates, Case Fatality[Title/Abstract])) OR (CFR Case Fatality Rate[Title/Abstract])) OR (Crude Death Rate[Title/Abstract])) OR (Crude Death Rates[Title/Abstract])) OR (Death Rate, Crude[Title/Abstract])) OR (Rate, Crude Death[Title/Abstract])) OR (Crude Mortality Rate[Title/Abstract])) OR (Crude Mortality Rates[Title/Abstract])) OR (Mortality Rate, Crude[Title/Abstract])) OR (Rate, Crude Mortality[Title/Abstract])) OR (Decline, Mortality[Title/Abstract])) OR (Mortality Declines[Title/Abstract])) OR (Mortality Decline[Title/Abstract])) OR (Mortality Determinants[Title/Abstract])) OR (Determinants, Mortality[Title/Abstract])) OR (Determinant, Mortality[Title/Abstract])) OR (Mortality Determinant[Title/Abstract])) OR (Mortality, Differential[Title/Abstract])) OR (Differential Mortality[Title/Abstract])) OR (Differential Mortalities[Title/Abstract])) OR (Age-Specific Death Rate[Title/Abstract])) OR (Age-Specific Death Rates[Title/Abstract])) OR (Death Rate, Age-Specific[Title/Abstract])) OR (Rate, Age-Specific Death[Title/Abstract])) OR (Age Specific Death Rate[Title/Abstract])) OR (Mortality, Excess[Title/Abstract])) OR (Excess Mortality[Title/Abstract])) OR (Excess Mortalities[Title/Abstract])) OR ("Mortality"[Mesh])) AND ((((((((((((((((((((((((((((((((((((((((Mortalities[Title/Abstract]) OR (Mortality Rate[Title/Abstract])) OR (Mortality Rates[Title/Abstract])) OR (Rate, Mortality[Title/Abstract])) OR (Death Rate[Title/Abstract])) OR (Death Rates[Title/Abstract])) OR (Rate, Death[Title/Abstract])) OR (Case Fatality Rate[Title/Abstract])) OR (Case Fatality Rates[Title/Abstract])) OR (Rate, Case Fatality[Title/Abstract])) OR (Rates, Case Fatality[Title/Abstract])) OR (CFR Case Fatality Rate[Title/Abstract])) OR (Crude Death Rate[Title/Abstract])) OR (Crude Death Rates[Title/Abstract])) OR (Death Rate, Crude[Title/Abstract])) OR (Rate, Crude Death[Title/Abstract])) OR (Crude Mortality Rate[Title/Abstract])) OR (Crude Mortality Rates[Title/Abstract])) OR (Mortality Rate, Crude[Title/Abstract])) OR (Rate, Crude Mortality[Title/Abstract])) OR (Decline, Mortality[Title/Abstract])) OR (Mortality Declines[Title/Abstract])) OR (Mortality Decline[Title/Abstract])) OR (Mortality Determinants[Title/Abstract])) OR (Determinants, Mortality[Title/Abstract])) OR (Determinant, Mortality[Title/Abstract]))** | 42 | 22:42:57 |

| | | | | | |
|---|---|---|---|---|---|
| | | | OR (Mortality Determinant[Title/Abstract])) OR (Mortality, Differential[Title/Abstract])) OR (Differential Mortality[Title/Abstract])) OR (Differential Mortalities[Title/Abstract])) OR (Age-Specific Death Rate[Title/Abstract])) OR (Age-Specific Death Rates[Title/Abstract])) OR (Death Rate, Age-Specific[Title/Abstract])) OR (Rate, Age-Specific Death[Title/Abstract])) OR (Age Specific Death Rate[Title/Abstract])) OR (Mortality, Excess[Title/Abstract])) OR (Excess Mortality[Title/Abstract])) OR (Excess Mortalities[Title/Abstract])) OR ("Mortality"[Mesh]))) AND ((((((((((Chronic Obstructive Lung Disease) OR (Chronic Obstructive Pulmonary Diseases)) OR (COAD)) OR (COPD)) OR (Chronic Obstructive Airway Disease)) OR (Chronic Obstructive Pulmonary Disease)) OR (Airflow Obstruction, Chronic)) OR (Airflow Obstructions, Chronic)) OR (Chronic Airflow Obstructions)) OR (Chronic Airflow Obstruction)) OR ("Pulmonary Disease, Chronic Obstructive"[Mesh]))) AND ((((((Lymphocyte) OR (Lymphoid Cells)) OR (Cell, Lymphoid)) OR (Cells, Lymphoid)) OR (Lymphoid Cell)) OR ("Lymphocytes"[Mesh]))) AND ((((((((((((((Neutrophil[Title/Abstract]) OR (Leukocytes, Polymorphonuclear[Title/Abstract])) OR (Leukocyte, Polymorphonuclear[Title/Abstract])) OR (Polymorphonuclear Leukocyte[Title/Abstract])) OR (Polymorphonuclear Leukocytes[Title/Abstract])) OR (Polymorphonuclear Neutrophils[Title/Abstract])) OR (Neutrophil, Polymorphonuclear[Title/Abstract])) OR (Polymorphonuclear Neutrophil[Title/Abstract])) OR (LE Cells[Title/Abstract])) OR (Cell, LE[Title/Abstract])) OR (LE Cel[Title/Abstract])) OR (Neutrophil Band Cells[Title/Abstract])) OR (Band Cell, Neutrophil[Title/Abstract])) OR (Neutrophil Band Cell[Title/Abstract])) OR ("Neutrophils"[Mesh])) | | |

History and Search Details

Download Delete

| Search | Actions | Details | Query | Results | Time |
|---|---|---|---|---|---|
| #11 | ••• | > | Search: (((((((((((((((((((((Neutrophils) OR (Neutrophil)) OR (Leukocytes)) OR (Polymorphonuclear)) OR (Polymorphonuclear)) OR (Polymorphonuclear Leukocyte)) OR (Polymorphonuclear Leukocytes)) OR (Polymorphonuclear Neutrophils)) OR (Neutrophil)) OR (Polymorphonuclear)) OR (Polymorphonuclear Neutrophil)) OR (LE Cells)) OR (Cell, LE)) OR (LE Cell)) OR (Neutrophil Band Cells)) OR (Band Cell)) OR (Neutrophil)) OR (Neutrophil Band Cell)) AND ((((((Lymphocytes) OR (Lymphocyte)) OR (Lymphoid Cells)) OR (Cell, Lymphoid)) OR (Cells, Lymphoid)) OR (Lymphoid Cell))) AND (ratio)) AND ((((((((((Chronic Obstructive Lung Disease) OR (Chronic Obstructive Pulmonary Diseases)) OR (COAD)) OR (COPD)) OR (Chronic Obstructive Airway Disease)) OR (Chronic Obstructive Pulmonary Disease)) OR (Airflow Obstruction, Chronic)) OR (Airflow Obstructions, Chronic)) OR (Chronic Airflow Obstructions)) OR (Chronic Airflow Obstruction))) AND (((((((((((((((((((((((((((((((((((((((Mortality) OR (Mortalities)) OR (Mortality Rate)) OR (Mortality Rates)) OR (Rate, Mortality)) OR (Death Rate)) OR (Death Rates)) OR (Rate, Death)) OR (Case Fatality Rate)) OR (Case Fatality Rates)) OR (Rate, Case Fatality)) OR (Rates, Case Fatality)) OR (CFR Case Fatality Rate)) OR (Crude Death Rate)) OR (Crude Death Rates)) OR (Crude Death Rates)) OR (Rate, Crude Death)) OR (Crude Mortality Rate)) OR (Crude Mortality Rates)) OR (Mortality Rate, Crude)) OR (Rate, Crude Mortality)) OR (Decline, Mortality)) OR (Mortality Declines)) OR (Mortality Decline)) OR (Mortality Determinants)) OR (Determinants, Mortality)) OR (Determinant, Mortality)) OR (Mortality Determinant)) OR (Mortality, Differential)) OR (Differential Mortality)) OR (Differential Mortalities)) OR (Age-Specific Death Rate)) OR (Age-Specific Death Rates)) OR (Death Rate, Age-Specific)) OR (Rate, Age-Specific Death)) OR (Age Specific Death Rate)) OR (Mortality, Excess)) OR (Excess Mortality)) OR (Excess Mortalities)) Filters: **from 2024 - 2025** | 28 | 08:53:18 |

OVID: Embase:718

Ovid® My Account Support & Training H

Search Journals Books My Workspace Visible Body Multimedia

▼ Search History (5)

| # ▲ | Searches | Results | Type |
|---|---|---|---|
| 1 | Neutrophil.af. or Leukocytes, Polymorphonuclea.ab. or Leukocyte, Polymorphonuclear.ab. or Polymorphonuclear Leukocyte.ab. or Polymorphonuclear Leukocytes.ab. or Polymorphonuclear Neutrophils.ab. or Neutrophil, Polymorphonuclear.ab. or Polymorphonuclear Neutrophil.ab. or LE Cells.ab. or Cell, LE.ab. or LE Cell.ab. or Neutrophil Band Cells.ab. or Band Cell, Neutrophil.ab. or Neutrophil Band Cell.ab. | 345054 | Advanced |
| 2 | (Lymphocyte or Lymphoid Cells or Cell, Lymphoid or Cells, Lymphoid or Lymphoid Cell).af. | 1164546 | Advanced |
| 3 | (Chronic Obstructive Lung Disease or Chronic Obstructive Pulmonary Diseases or COAD or COPD or Chronic Obstructive Airway Disease or Chronic Obstructive Pulmonary Disease or Airflow Obstruction, Chronic or Airflow Obstructions, Chronic or Chronic Airflow Obstructions or Chronic Airflow Obstruction).af. | 213734 | Advanced |
| 4 | Mortalities.af. or Mortality Rate.ab. or Mortality Rates.ab. or Rate, Mortality.ab. or Death Rate.ab. or Death Rates.ab. or Rate, Death.ab. or Case Fatality Rate.ab. or Case Fatality Rates.ab. or Rate, Case Fatality.ab. or CFR Case Fatality Rate.ab. or Crude Death Rate.ab. or Crude Death Rates.ab. or Death Rate, Crude.ab. or Rate, Crude Death.ab. or Crude Mortality Rate.ab. or Crude Mortality Rates.ab. or Mortality Rate, Crude.ab. or Rate, Crude Mortality.ab. or Decline, Mortality.ab. or Mortality Declines.ab. or Mortality Decline.ab. or Mortality Determinants.ab. or Determinants, Mortality.ab. or Determinant, Mortality.ab. or Mortality Determinant.ab. or Mortality, Differential.ab. or Differential Mortality.ab. or Differential Mortalities.ab. or Age-Specific Death Rate.ab. or Age-Specific Death Rates.ab. or Death Rate, Age-Specific.ab. or Rate, Age-Specific Death.ab. or Age Specific Death Rate.ab. or Mortality, Excess.ab. or Excess Mortality.ab. or Excess Mortalities.ab. or Rates, Case Fatality.ab. or Mortality.ab. | 1489778 | Advanced |
| 5 | 1 and 2 and 3 and 4 | 572 | Advanced |

Save Remove Combine with: AND OR

Ovid My Account My PayPerView Support & Training Help Feedback Log Off

Search Journals Books Multimedia My Workspace What's New

Search History (8) View Saved

| # ▼ | Searches | Results | Type | Actions | Annotations |
|---|---|---|---|---|---|
| 8 | limit 7 to yr="2024 - 2025" | 146 | Advanced | Display Results More | |
| 7 | 4 and 5 and 6 | 586 | Advanced | Display Results More | |
| 6 | (Mortality or Mortalities or Mortality Rate or Mortality Rates or Rate, Mortality or Death Rate or Death Rates or Rate, Death or Case Fatality Rate or Case Fatality Rates or Rate, Case Fatality or Rates, Case Fatality or CFR Case Fatality Rate or Crude Death Rate or Crude Death Rates or Crude Death Rates or Rate, Crude Death or Crude Mortality Rate or Crude Mortality Rates or Mortality Rate, Crude or Rate, Crude Mortality or Decline, Mortality or Mortality Declines or Mortality Decline or Mortality Determinants or Determinants, Mortality or Determinant, Mortality or Mortality Determinant or Mortality, Differential or Differential Mortality or Differential Mortalities or Age-Specific Death Rate or Age-Specific Death Rates or Death Rate, Age-Specific or Rate, Age-Specific Death or Age Specific Death Rate or Mortality, Excess or Excess Mortality or Excess Mortalities).af. | 2136585 | Advanced | Display Results More | |
| 5 | (Chronic Obstructive Lung Disease or Chronic Obstructive Pulmonary Diseases or COAD or COPD or Chronic Obstructive Airway Disease or Chronic Obstructive Pulmonary Disease or Airflow Obstruction, Chronic or Airflow Obstructions, Chronic or Chronic Airflow Obstructions or Chronic Airflow Obstruction).af. | 231818 | Advanced | Display Results More | |

Save Remove Combine with: AND OR Expand

OVID : COCHRANE 3

THIS LEGACY OVID INTERFACE WILL GO AWAY ON MARCH 27, 2024 - SWITCH TO THE CURRENT OVID INTERFACE

Ovid® Wolters Kluwer

My Account Support & Training Help Feedback Logoff

Search Journals Books Multimedia My Workspace BioDigital Human What's New

▼ Search History (5) View Saved

| # ▲ | Searches | Results | Type | Actions | Annotations |
|---|---|---|---|---|---|
| 1 | (Neutrophil or Leukocytes, Polymorphonuclear or Leukocyte, Polymorphonuclear or Polymorphonuclear Leukocyte or Polymorphonuclear Leukocytes or Polymorphonuclear Neutrophils or Neutrophil, Polymorphonuclear or Polymorphonuclear Neutrophil or LE Cells or Cell, LE or LE Cell or Neutrophil Band Cells or Band Cell, Neutrophil or Neutrophil Band Cell).af. | 11638 | Advanced | Display Results More | |
| 2 | (Lymphocyte or Lymphoid Cells or Cell, Lymphoid or Cells, Lymphoid or Lymphoid Cell).af. | 20441 | Advanced | Display Results More | |
| 3 | (Chronic Obstructive Lung Disease or Chronic Obstructive Pulmonary Diseases or COAD or COPD or Chronic Obstructive Airway Disease or Chronic Obstructive Pulmonary Disease or Airflow Obstruction, Chronic or Airflow Obstructions, Chronic or Chronic Airflow Obstructions or Chronic Airflow Obstruction).af. | 23652 | Advanced | Display Results More | |
| 4 | Mortalities.af. or Mortality Rate.ab. or Mortality Rates.ab. or Rate, Mortality.ab. or Death Rate.ab. or Death Rates.ab. or Rate, Death.ab. or Case Fatality Rate.ab. or Case Fatality Rates.ab. or Rate, Case Fatality.ab. or Rates, Case Fatality.ab. or CFR Case Fatality Rate.ab. or Crude Death Rate.ab. or Crude Death Rates.ab. or Death Rate, Crude.ab. or Rate, Crude Death.ab. or Crude Mortality Rate.ab. or Crude Mortality Rates.ab. or Mortality Rate, Crude.ab. or Rate, Crude Mortality.ab. or Decline, Mortality.ab. or Mortality Declines.ab. or Mortality Decline.ab. or Mortality Determinants.ab. or Determinants, Mortality.ab. or Determinant, Mortality.ab. or Mortality Determinant.ab. or Mortality, Differential.ab. or Differential Mortality.ab. or Differential Mortalities.ab. or Age-Specific Death Rate.ab. or Age-Specific Death Rates.ab. or Death Rate, Age-Specific.ab. or Rate, Age-Specific Death.ab. or Age Specific Death Rate.ab. or Mortality, Excess.ab. or Excess Mortality.ab. or Excess Mortalities.ab. | 13090 | Advanced | Display Results More | |
| 5 | 1 and 2 and 3 and 4 | 2 | Advanced | Display Results More | |

Contract

Limit a Search

| # ▲ | Searches | Results | Type |
|---|---|---|---|
| 1 | ((Neutrophils or Neutrophil or Leukocytes or Polymorphonuclear or Polymorphonuclear or Polymorphonuclear Leukocyte or Polymorphonuclear Leukocytes or Polymorphonuclear Neutrophils or Neutrophil or Polymorphonuclear or Polymorphonuclear Neutrophil or LE Cells or Cell, LE or LE Cell or Neutrophil Band Cells or Band Cell or Neutrophil or Neutrophil Band Cell) and (Lymphocytes or Lymphocyte or Lymphoid Cells or Cell, Lymphoid or Cells, Lymphoid or Lymphoid Cell) and ratio and (Chronic Obstructive Lung Disease or Chronic Obstructive Pulmonary Diseases or COAD or COPD or Chronic Obstructive Airway Disease or Chronic Obstructive Pulmonary Disease or Airflow Obstruction, Chronic or Airflow Obstructions, Chronic or Chronic Airflow Obstructions or Chronic Airflow Obstruction) and (Mortality or Mortalities or Mortality Rate or Mortality Rates or Rate, Mortality or Death Rate or Death Rates or Rate, Death or Case Fatality Rate or Case Fatality Rates or Rate, Case Fatality or Rates, Case Fatality or CFR Case Fatality Rate or Crude Death Rate or Crude Death Rates or Crude Death Rates or Rate, Crude Death or Crude Mortality Rate or Crude Mortality Rates or Mortality Rate, Crude or Rate, Crude Mortality or Decline, Mortality or Mortality Declines or Mortality Decline or Mortality Determinants or Determinants, Mortality or Determinant, Mortality or Mortality Determinant or Mortality, Differential or Differential Mortality or Differential Mortalities or Age-Specific Death Rate or Age-Specific Death Rates or Death Rate, Age-Specific or Rate, Age-Specific Death or Age Specific Death Rate or Mortality, Excess or Excess Mortality or Excess Mortalities)).af. | 11 | Advanced |
| 2 | limit 1 to yr="2024 - 2025" | 1 | Advanced |

WOS :170

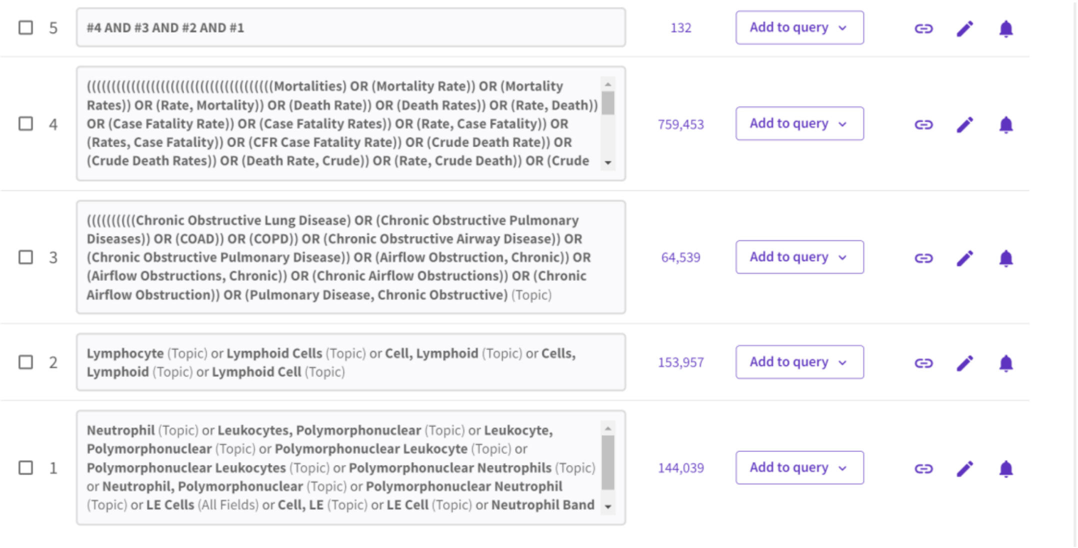
5
#4 AND #3 AND #2 AND #1
132
Add to query
4
((((((((((((((((((((((((((((((((((((((Mortalities) OR (Mortality Rate)) OR (Mortality Rates)) OR (Rate, Mortality)) OR (Death Rate)) OR (Death Rates)) OR (Rate, Death)) OR (Case Fatality Rate)) OR (Case Fatality Rates)) OR (Rate, Case Fatality)) OR (Rates, Case Fatality)) OR (CFR Case Fatality Rate)) OR (Crude Death Rate)) OR (Crude Death Rates)) OR (Death Rate, Crude)) OR (Rate, Crude Death)) OR (Crude
759,453
Add to query
3
(((((((((Chronic Obstructive Lung Disease) OR (Chronic Obstructive Pulmonary Diseases)) OR (COAD)) OR (COPD)) OR (Chronic Obstructive Airway Disease)) OR (Chronic Obstructive Pulmonary Disease)) OR (Airflow Obstruction, Chronic)) OR (Airflow Obstructions, Chronic)) OR (Chronic Airflow Obstructions)) OR (Chronic Airflow Obstruction)) OR (Pulmonary Disease, Chronic Obstructive) (Topic)
64,539
Add to query
2
Lymphocyte (Topic) or Lymphoid Cells (Topic) or Cell, Lymphoid (Topic) or Cells, Lymphoid (Topic) or Lymphoid Cell (Topic)
153,957
Add to query
1
Neutrophil (Topic) or Leukocytes, Polymorphonuclear (Topic) or Leukocyte, Polymorphonuclear (Topic) or Polymorphonuclear Leukocyte (Topic) or Polymorphonuclear Leukocytes (Topic) or Polymorphonuclear Neutrophils (Topic) or Neutrophil, Polymorphonuclear (Topic) or Polymorphonuclear Neutrophil (Topic) or LE Cells (All Fields) or Cell, LE (Topic) or LE Cell (Topic) or Neutrophil Band
144,039
Add to query

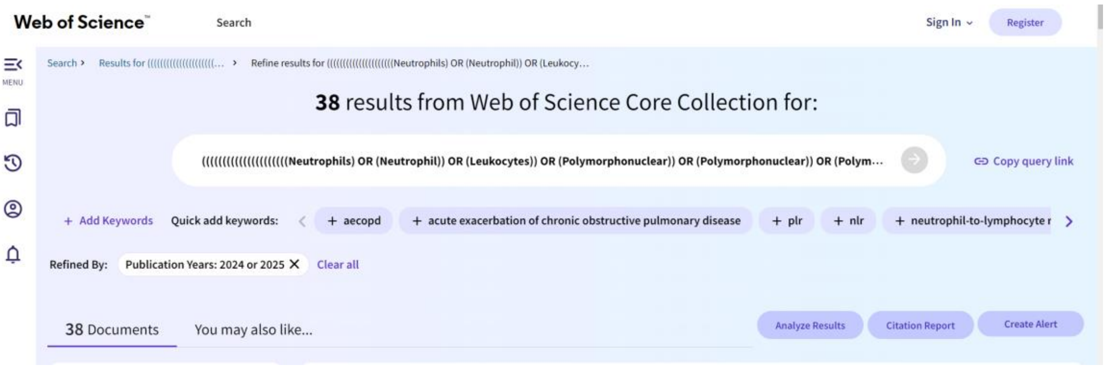
Web of Science
Search
Sign In
Register
MENU
Search › Results for ((((((((((((((((((... › Refine results for ((((((((((((((((((Neutrophils) OR (Neutrophil)) OR (Leukocy...
38 results from Web of Science Core Collection for:
((((((((((((((((((Neutrophils) OR (Neutrophil)) OR (Leukocytes)) OR (Polymorphonuclear)) OR (Polymorphonuclear)) OR (Polym...
Copy query link
+ Add Keywords
Quick add keywords:
+ aecopd
+ acute exacerbation of chronic obstructive pulmonary disease
+ plr
+ nlr
+ neutrophil-to-lymphocyte r
Refined By:
Publication Years: 2024 or 2025
Clear all
38 Documents
You may also like...
Analyze Results
Citation Report
Create Alert
